# Molecular Analyses Reveal Unexpected Genetic Structure in Iberian Ibex Populations

**DOI:** 10.1371/journal.pone.0170827

**Published:** 2017-01-30

**Authors:** Samer Angelone-Alasaad, Iris Biebach, Jesús M. Pérez, Ramón C. Soriguer, José E. Granados

**Affiliations:** 1 Institute of Evolutionary Biology and Environmental Studies (IEU), University of Zürich, Winterthurerstrasse 190, Zürich, Switzerland; 2 Estación Biológica de Doñana, Consejo Superior de Investigaciones Científicas (CSIC), Avda. Américo Vespucio s/n Sevilla, Spain; 3 Departamento de Biología Animal, Biología Vegetal y Ecología, Universidad de Jaén, Campus Las Lagunillas, s/n, Jaén, Spain; 4 Espacio Natural de Sierra Nevada, Carretera Antigua de Sierra Nevada, km 7, Pinos Genil, Granada, Spain; Embrapa, BRAZIL

## Abstract

**Background:**

Genetic differentiation in historically connected populations could be the result of genetic drift or adaptation, two processes that imply a need for differing strategies in population management. The aim of our study was to use neutral genetic markers to characterize *C*. *pyrenaica* populations genetically and examine results in terms of (i) demographic history, (ii) subspecific classification and (iii) the implications for the management of Iberian ibex.

**Methodology/Principal Findings:**

We used 30 neutral microsatellite markers from 333 Iberian ibex to explore genetic diversity in the three main Iberian ibex populations in Spain corresponding to the two persisting subspecies (*victoria* and *hispanica*). Our molecular analyses detected recent genetic bottlenecks in all the studied populations, a finding that coincides with the documented demographic decline in *C*. *pyrenaica* in recent decades. Genetic divergence between the two *C*. *pyrenaica* subspecies (*hispanica* and *victoriae*) was substantial (*F*_*ST*_ between 0.39 and 0.47). Unexpectedly, we found similarly high genetic differentiation between two populations (Sierra Nevada and Maestrazgo) belonging to the subspecies *hispanica*. The genetic pattern identified in our study could be the result of strong genetic drift due to the severe genetic bottlenecks in the studied populations, caused in turn by the progressive destruction of natural habitat, disease epidemics and/or uncontrolled hunting.

**Conclusions:**

Previous *Capra pyrenaica* conservation decision-making was based on the clear distinction between the two subspecies (*victoriae* and *hispanica*); yet our paper raises questions about the usefulness for conservation plans of the distinction between these subspecies.

## Introduction

In the past century many animal populations were greatly reduced in size or driven to extinction by human activities such as habitat destruction or hunting [1]. Consequently, the gene flow between remaining populations was reduced or interrupted. Increased genetic drift in populations leads to genetic differentiation between populations (e.g. [2]). However, genetic differentiation can also arise in populations due to adaptation to different habitats [3]. Each of these two causes of genetic differentiation has different implications for population management. If genetic drift is the cause of genetic differentiation, gene flow between populations should be re-established to increase genetic variation, decrease potential inbreeding and thus increase the fitness of the populations. However, if adaptation is the cause of differentiation, populations should be kept separate to prevent the introduction of maladapted genes [4]. As a result, the decision regarding how to manage formerly connected populations is an important challenge for conservation biologists [5]. Genetic analysis and knowledge of the history of populations are both helpful in this decision-making process.

**Fig 1:**
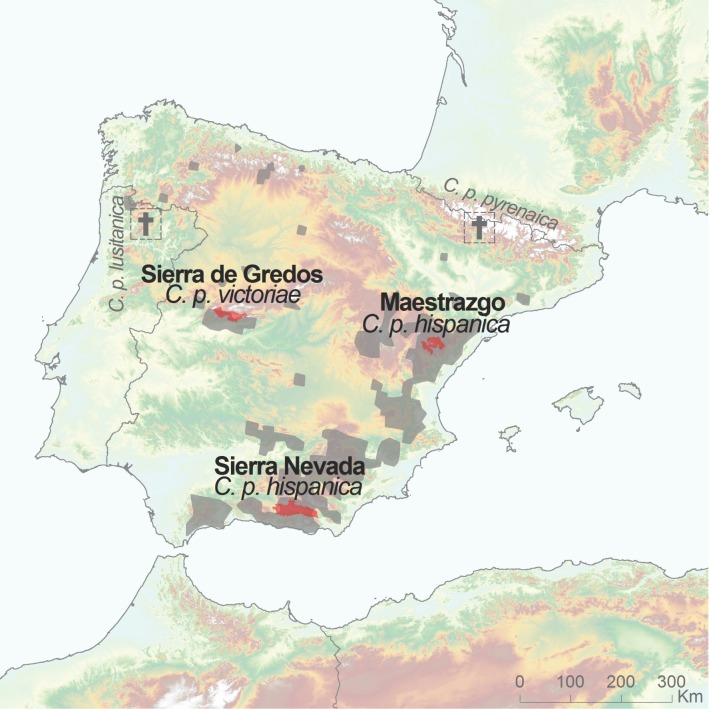
Map of the Iberian Peninsula showing the current Iberian ibex (*Capra pyrenaica*) distribution (grey colour) with the three studied populations highlighted in red. Both the *C*. *p*. *pyrenaica* and *C*. *p*. *lusitanica* subspecies are extinct.

The Iberian ibex *Capra pyrenaica* [6] is endemic to the Iberian Peninsula. Four subspecies of this ungulate have been recognized based on coat colour and horn morphology [7–10], two of which, *C*. *p*. *lusitanica*, formerly found in northern Portugal and southern Galicia, and *C*. *p*. *pyrenaica*, once present in the Pyrenees, are now extinct [11], [12]. The remaining two subspecies, *C*. *p*. *hispanica*, found in the south and east of the Iberian Peninsula, and *C*. *p*. *victoriae*, found mainly in central and northwestern Spain, have an allopatric distribution in the Iberian Peninsula [13], [14] (Fig 1).

However, the subspecific classification of the Iberian ibex is based purely on morphological traits and, given that relevant biometric data are scarce and incomplete [15], [16], this classification has been widely questioned [16–18]. Analyses using data from mitochondrial DNA sequences [19] add further doubts to this subspecific classification [20]. Moreover, because of its value as a game animal, this species has been the object of numerous translocation and restocking programs, many poorly documented [20], [21], that may have led to an admixture of animals from different geographical regions. To date, no comprehensive analysis combining genetic and morphological data has ever been carried out [20], [22].

During the nineteenth century, the Iberian ibex underwent a significant demographic decline due to the progressive destruction of its natural habitat, disease epidemics and uncontrolled hunting [23]. At the beginning of the twentieth century, however, a number of conservation programs helped Iberian ibex populations begin to recover [12], [24], [25]. Currently, the two persisting subspecies are both recovering and expanding [26], with *C*. *p*. *hispanica* today more widely distributed than *C*. *p*. *victoriae*, whose populations are fewer and smaller in extent [14]. Recently a new population of *C*. *p*. *victoria* has been established in Portugal [27].

The aim of our study was to use neutral genetic markers to characterize genetically *C*. *pyrenaica* populations and discuss the results in terms of (i) the demographic history of these populations, (ii) their subspecific classification and (iii) the implications for Iberian ibex management.

## Methods

### Samples collection and DNA extraction

Between 2003 and 2008, we collected 333 Iberian ibex samples from the three main Spanish populations: 69 *C*. *p*. *hispanica* from Maestrazgo Natural Park (north-eastern Spain), 238 *C*. *p*. *hispanica* from Sierra Nevada Natural Space (southern Spain), and 26 *C*. *p*. *victoriae* from Sierra de Gredos (north-central Spain) (Fig 1). Map was prepared using political boundaries and USGS (These data are distributed by the Land Processes Distributed Active Archive Center (LP DAAC), located at USGS/EROS, Sioux Falls, SD. http://lpdaac.usgs.gov) [28]. The spatial distributions of *Capara pyrenaica* is adapted from [12].

Samples consisted of tissue (small biopsy from the ears) obtained from deceased legally hunted animals, or from animals culled by park rangers as part of wildlife management plans aimed especially at combating outbreaks of diseases. This study was approved by the Ministry of Agriculture, Fishery and Environment of the Andalusian government (Junta de Andalucía). Sampling procedures were sanctioned as part of the application for permits for the fieldwork, which did not affect any endangered or protected species.

The Maestrazgo population of *C*. *p*. *hispanica* may have suffered from two consecutive demographic bottlenecks, one in the period after the Spanish Civil War (1940–1945) and the other in the 1960s, when the population fell to just 30 animals and almost became extinct [17]. However, conservation programs [19] enabled this population to recover to close to 7,000 individuals by 1998 [12].

The Sierra Nevada Iberian ibex population was estimated at 600 individuals in the 1960s [17] and is nowadays the largest Iberian ibex population and the main one of subspecies *C*. *p*. *hispanica* (close to 16,000 individuals [12]). This population is considered to be the most genetically diverse [19].

The Sierra de Gredos population of *C*. *p*. *victoriae* numbered around 50 individuals in 1895 and just 12 individuals in 1905 [17]. A conservation program, which started in that year, helped this population to recover and by the 1960s it had grown to almost 2,800 individuals [17]. Nowadays, the Gredos population is the second largest *C*. *pyrenaica* population and the main population of subspecies *C*. *p*. *victoriae* (close to 8,000 individuals; [12]). Tissue samples were taken from legally hunted animals in 2004–2007 and were stored in 100% ethanol at -20°C before genomic DNA extraction with a commercial kit (BioSprint 96 and QIAamp DNA Mini Kit; QIAGEN).

### Microsatellite genotyping

A total of 30 microsatellites, previously tested on the Alpine ibex [2], [29], were used in our study, with the same multiplex panels and thermal profiles as described in Biebach & Keller [2] (see S1 Table). PCR products were analyzed on an ABI 3100 Avant automated sequencer. Allele sizes and genotypes were determined using GeneMapper 3.7 (Applied Biosystems), followed by manual proofreading. In each PCR run a negative control (no DNA) sample was included. Genotypes of low quality were repeated or not scored (for more details, see S1 Table).

### Statistical analyses

Expected (*H*_*E*_) and observed (*H*_*O*_) heterozygosity, and deviations from the Hardy-Weinberg equilibrium (HWE) were calculated using Genepop (v.3.4; [30]). Fisher’s exact tests and sequential Bonferroni corrections were used to evaluate deviations from HWE. Linkage disequilibrium (LD) between pairs of microsatellite loci within populations was estimated with Arlequin 3.11 [31].

Genetic divergence in the three Iberian ibex populations was assessed using pairwise *F*_*ST*_ based on allele frequencies as implemented in Arlequin 3.11.

We estimated standardized allelic richness in FSTAT version 2–9.3. [32] to account for the substantially different sample sizes of the studied Iberian ibex populations.

The relationships between the *C*. *pyrenaica* populations were assessed using a dendrogram calculated using Nei's standard genetic distance based on allele frequencies [33] constructed with the POPGENE software package [34]. Additionally, we used two further methods, namely:

1. The clustering program INSTRUCT v1.1 [35]: population structure was inferred for K (number of clusters) = 1 to 8, with 10 chains for each K. The Gelman–Rubin statistic (Rc) was used to check for convergence [36]. The optimal K was inferred using the deviance information criterion (DIC: [37]).
2. The DAPC [38] software implemented in the *adegenet* R package [39] to identify the number of clusters. The maximum number of clusters was set at eight. The number of clusters was determined using the Bayesian Information Criteria (BIC) following the k-means algorithm.

In recently bottlenecked populations, observed heterozygosity is higher than expected given the number of alleles in the population if the population is at mutation-drift equilibrium [40]. This heterozygosity excess was used to test for the genetic signature of bottlenecks in Iberian ibex as implemented in Bottleneck (v.1.2.02; [41]). We used 10,000 iterations for each of the three tests (sign test, standardized differences test and Wilcoxon sign-rank test) per population. We used the intermediate two-phase mutation model (TPM) [41], which has been shown to deliver the most realistic results for the typical mutational events of microsatellite loci [42]. The heterozygosity excess tested by means of BOTTLENECK refers to “the fact that heterozygosity computed from a sample of genes is larger than the heterozygosity expected from the number of alleles found in the sample if the population were at mutation-drift equilibrium (HEQ)” [40]. All input file preparations were made using Convert (v. 1.31; [43]).

## Results

Of the 30 microsatellite loci, OarFCB48 was monomorphic in all three Iberian ibex populations, while OarVH34 deviated from HWE (*p*< 0.001) in all populations. Hence, both microsatellite markers were excluded from further analysis. Significant deviation from HWE after a Bonferroni correction [44] was present in SR-CRSP24 (*p* = 0.001), BM4505 (*p* = 0.034), MILSTS76 (*p* = 0.005) and URB058 (*p* = 0.017) in Maestrazgo Natural Park, and in BM4208 (*p* = 0.005), SR-CRSP24 (*p*< 0.001), BM415 (*p* = 0.007), MAF36 (*p* = 0.015), CSSM47 (*p* = 0.016), HAUT27 (*p*< 0.001) and GLA10 (*p* = 0.029) in Sierra Nevada Natural Space. The pairwise comparisons revealed that 2.52% of all pairs of microsatellite loci were in linkage disequilibrium, after correction for multiple tests.

A total of 140 alleles were detected in the 28 microsatellite loci retained in the analyses: 67 alleles in Sierra de Gredos, 75 in Maestrazgo Natural Park, and 91 in Sierra Nevada Natural Space (Table 1). Sierra Nevada Natural Space has slightly greater standardized allelic richness than the Maestrazgo Natural Park population but, contrary to expectations, has lower expected heterozygosity than the Maestrazgo Natural Park population.

**Table 1:** Number of alleles, standardized allelic richness, mean observed heterozygosity (*H*_*O*_), expected heterozygosity (*H*_*E*_) and *F*_IS_ (estimate of deviation from random mating) per population of the two studied *C*. *pyrenaica* subspecies corresponding to three Iberian ibex populations in Spain.

	N Animals	N Alleles	Standardized Allelic Richness	*H*_*O*_	*H*_*E*_	*F*_*is*_
Sierra de Gredos	26	2.39	2.36	0.36	0.35	-0.03
Maestrazgo National Park	69	2.68	2.54	0.41	0.43	0.06
Sierra Nevada National Space	238	3.25	2.55	0.37	0.39	0.05

The mean *F*_IS_ per population was not significantly different from 0 and thus there was no deviation from random mating in the study populations (Table 1).

In all, 71 of the 140 alleles in all loci were private alleles in one of the populations: 19 (26.8%) in *C*. *p*. *victoriae* in Sierra de Gredos, 20 (28.2%) in *C*. *p*. *hispanica* in Maestrazgo Natural Park, and 32 (45%) in *C*. *p*. *hispanica* in Sierra Nevada Natural Space. The number of private alleles per locus ranged from 0 (BM1818) to six (MAF36). Interestingly, the Sierra Nevada Natural Space population of *C*. *p*. *hispanica* had the highest proportion of private alleles, even higher than the *C*. *p*. *victoriae* population in Sierra de Gredos.

The analysis of genetic divergence revealed strong genetic differentiation in the three populations. Unexpectedly, the *F*_*ST*_ value between the two *C*. *p*. *hispanica* populations (*F*_*ST*_ = 0.38; p<0.001) was of similar magnitude as the *F*_*ST*_ values between *C*. *p*. *victoriae* and *C*. *p*. *hispanica* (0.39 and 0.47, respectively; p<0.001 for both; Table 2).

**Table 2:** Matrix of population pairwise FST (below diagonal) and significance level (above diagonal) for each pairwise comparison of the three Iberian ibex populations from Spain.

	Sierra de Gredos	Maestrazgo Natural Park	Sierra Nevada Natural Space
Sierra de Gredos (*C*. *p*. *victoriae*)		<0.001*	<0.001*
Maestrazgo Natural Park (*C*. *p*. *hispanica*)	0.3862		<0.001*
Sierra Nevada Natural Space (*C*. *p*. *hispanica*)	0.4748	0.3841	

* Statistically significant.

According both to DAPC and to INSTRUCT, K = 3 represented the optimal number of clusters for describing the clustering of these three ibex populations.

**Fig 2:**
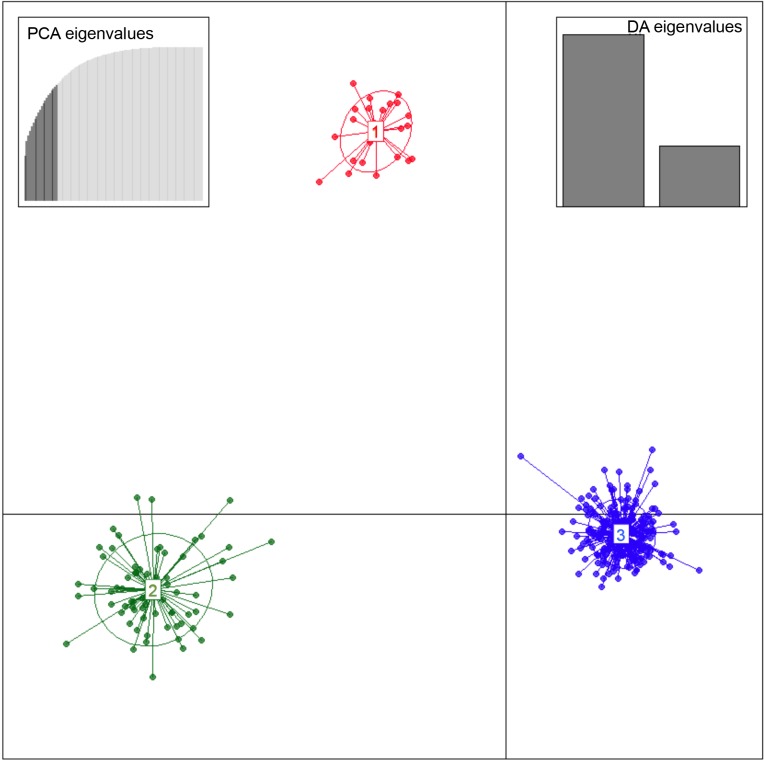
Results of the DAPC analysis of the three main ibex populations in Spain when the number of genetic clusters is 3 (K = 3). 1: Sierra de Gredos (*C*. *p*. *victoriae*). 2: Maestrazgo Natural Park (*C*. *p*. *hispanica*). 3: Sierra Nevada Natural Space (*C*. *p*. *hispanica*).

**Fig 3:**
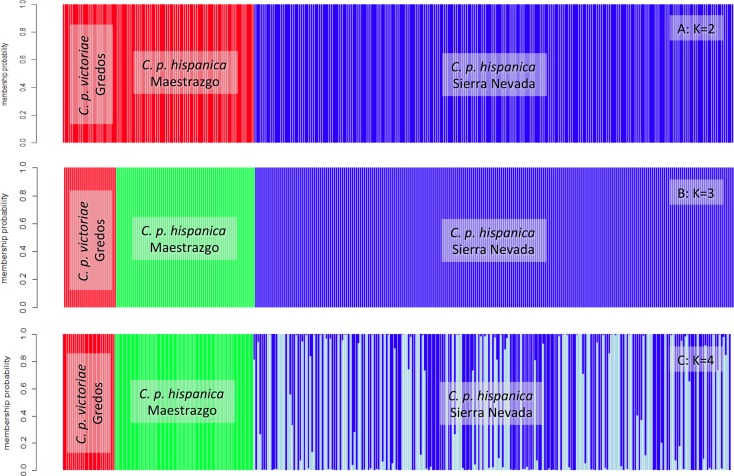
Bar plots of the proportion of individual variation in 333 Iberian ibex from the three main ibex populations in Spain assigned to given genetic clusters in INSTRUCT, with two (A: K = 2), three (B: K = 3) and four (C: K = 4) clusters. Each cluster is represented by a different colour.

When applying K = 3, Iberian ibex were separated as per the three studied populations (Sierra de Gredos, Maestrazgo Natural Park and Sierra Nevada Natural Space). With K = 2, *C*. *p*. *hispanica* from Sierra Nevada Natural Space formed one cluster and the other two populations, *C*. *p*. *victoriae* from Sierra de Gredos and *C*. *p*. *hispanica* from Maestrazgo Natural Park, were assigned to another. For K>3 the three groups of K = 3 remained, but individuals from the *C*. *p*. *hispanica* population from Sierra Nevada Natural Space were assigned with different proportions to the additional clusters. There was no clear substructure in the population from Sierra Nevada because individuals were not assigned clearly either one of the clusters (Fig 2 & Fig 3).

**Fig 4:**
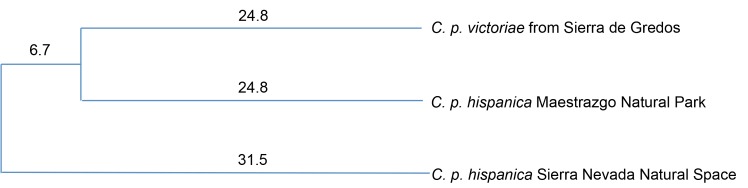
Unrooted dendrogram showing the genetic relationships (genetic divergence as a percentage %) in the three studied Iberian ibex populations based on Nei’s standard genetic distance [32].

The dendrogram based on Nei’s standard genetic distance (Fig 4) showed a closer genetic relationship between the Sierra de Gredos (*C*. *p*. *victoria*) and Maestrazgo Natural Park (*C*. *p*. *hispanica*) populations than between the two populations of *C*. *p*. *hispanica* (Maestrazgo Natural Park and Sierra Nevada Natural Space).

For the Maestrazgo Natural Park population, all three tests for a bottleneck (sign test, standardized differences test and Wilcoxon's signed rank test) detected significant heterozygosity excess compared to expectations if the population were at mutation-drift equilibrium. For the Sierra de Gredos population, two tests showed a significant result for a bottleneck, while the third test was just non-significant (sign test, p = 0.053). None of the tests indicated a bottleneck for the Sierra Nevada Natural Space population (Table 3).

**Table 3:** Parameters and results for bottleneck analyses used to detect significant reductions in effective population size in Iberian ibex from different populations in Spain. The parameters for heterozygosity (expected heterozygosity excess, heterozygosity excess and heterozygosity deficiency) refer to the number of loci.

	Bottleneck Test (TPM mutation model)		
	Sierra de Gredos	Maestrazgo Natural Park	Sierra Nevada Natural Space
Sign test	Expected heterozygosity excess = 11.68	Expected heterozygosity excess = 13.42	Expected heterozygosity excess = 13.93
	heterozygosity deficiency = 7	heterozygosity deficiency = 7	heterozygosity deficiency = 8
	heterozygosity excess = 16	heterozygosity excess = 18	heterozygosity excess = 18
	p = 0.05266	p = 0.04751	p = 0.07646
Standardized differences test	T2 = 2.419	T2 = 3.268	T2 = 0.956
	P = 0.00778	P = 0.00054	P = 0.16965
Wilcoxon's signed rank test	P = 0.00373	P = 0.00113	P = 0.12891

## Discussion

Different loci deviated from HWE in different populations. These deviations are more likely to be due to the subtle substructure of the Sierra Nevada and Maestrazgo populations than to the inherent characteristics of these loci; the possible exception is SR-CRSP24, which deviated from HWE in two of the three study populations and also deviated from HWE in some populations of Alpine ibex [2]. The found linkage disequilibrium in the three studied populations might be the result of bottlenecks [45] since these populations have low genetic variation like other populations such as the Alpine ibex that have experienced severe bottlenecks [2].

Historically *C*. *pyrenaica hispanica* in Sierra Nevada has experienced less severe bottlenecks than the other two populations. Therefore, in the Sierra Nevada population genetic drift was likely to be less than in the Maestrazgo and Sierra de Gredos populations. Genetic drift has a great effect on the proportion of private alleles [46] and seems to have shaped the number of private alleles in the studied *C*. *pyrenaica* populations. The Sierra Nevada population with the least severe bottleneck has the highest proportion of private alleles. The generally high amount of private alleles in all *C*. *pyrenaica* populations indicates a lack of gene flow within the three populations.

The significant genetic signature of a bottleneck in at least two of the three tests for the Maestrazgo and Sierra de Gredos populations matches the known severe demographic bottlenecks in these two populations, which were reduced to just 30 and 12 ibex, respectively, in the 1960s and at the beginning of the twentieth century [17]. The Sierra Nevada population–a low point of 600 individuals in the 1960s –experienced the least severe bottleneck. The absence of a genetic signature for a bottleneck in this population might be due to the low power of the test for this bottleneck. Assuming a ratio of the effective population size to census size of 0.34 [2] and a generation time of eight years [47], the power analysis of Cornuet and Luikart [40] predicts a detection probability for the bottleneck of between 20% and 40%, even with the reasonably high number of loci and samples used in the analysis. Over the coming centuries, when more ibex generations have come and gone, the power of this test should increase [40].

Our results from 28 neutral microsatellite loci highlighted unexpectedly high genetic differentiation between the populations of *C*. *p*. *hispanica* from Maestrazgo Natural Park and from Sierra Nevada, a finding that was not predicted because these populations belong to the same subspecies, *C*. *p*. *hispanica*. Furthermore, these two mountain ranges are connected, albeit over a total distance of around 800 km [20] (Fig 1). The fairly high number of private alleles in these two *C*. *p*. *hispanica* populations and the high genetic differentiation (*F*_*ST*_ = 0.3841) can only arise if there is only very limited gene flow between the populations and if genetic drift is occurring. The history of these populations and the bottleneck tests indicate that genetic drift is operating in each population, but more so in the Maestrazgo population.

We found as much genetic divergence between the Sierra Gredos (*C*. *p*. *victoria*) and Maestrazgo (*C*. *p*. *hispanica*) Iberian ibex populations as between the two populations of *C*. *p*. *hispanica*. The high *F*_*ST*_ values between these two subspecies confirm that they are genetically quite different. However, we detected as much genetic differentiation within the subspecies *C*. *p*. *hispanica* as between the two subspecies. This unexpected genetic structure of these three populations raises the question as to whether genetic differentiation between subspecies reflects adaptive differentiation or is, rather, the consequence of random genetic changes due to genetic drift. Research into the differences in adaptation between these subspecies as needed to clarify this issue.

The distinction between adaptation and genetic drift as a cause of genetic differentiation is relevant for the future management of Iberian ibex. The low genetic variation in the studied Iberian ibex populations suggests weak adaptive evolutionary potential, although this needs to be verified using more markers (e.g. SNPs) that are able to infer the adaptive potential better than the microsatellites [48]. The strong genetic drift experienced in the Iberian ibex populations indicates low effective population sizes and, in turn, potential high levels of inbreeding, which could reduce the short-term and long-term survival of these populations [49].

Admixture between genetically differentiated populations is a management option that can both decrease inbreeding and increase genetic variation in genetically impoverished populations, thereby enhancing the fitness of the population [50]. Admixing populations entails a risk of outbreeding depression due to adaptive differentiation from selection or fixation of chromosomal variants [51]. Nevertheless, admixed individuals of two populations have a low probability of outbreeding depression if the two populations were isolated less than 500 years ago, inhabit similar environments (or different ones for no more than 20 generations), and have no chromosomal differences [51]. Thus, identifying reasons for the marginal gene flow between the populations of *C*. *p*. *hispanica* in Maestrazgo and Sierra Nevada, and (re-)establishing gene flow between them should be a future management goal.

### Conclusions

The results of this study question the usefulness of the *Capra pyrenaica* subspecies denomination and the current avoidance of admixtures of these subspecies in conservation plans [12]. For instance, current management plans for the Iberian ibex include the reintroduction of the subspecies *C*. *p*. *victoriae* from Sierra de Guadarrama into the Pyrenees where the subspecies *C*. *p*. *pyrenaica* is today extinct [52].

However, a study of the mitochondrial DNA of seven *Capra pyrenaica* populations (including the three populations used in this study) found that extant *Capra pyrenaica* populations are as equally genetically distant as the extinct subspecies. This has led Manceau et al. [19] to suggest admixing individuals from diverse *Capra pyrenaica* populations to establish a new population with high genetic diversity in which selection can begin to operate. The results of our study with nuclear markers point in the same direction: the founding of a new Pyrenees population with ibex from all the remaining Iberian ibex populations–regardless of their subspecies–would enhance their adaptive potential in the new environment and only entail a low risk of outbreeding depression.

Translocation of individuals from a genetically different population to increase the fitness of a recipient population has been successfully accomplished in some genetically imperilled populations [53]. The Florida panther (*Puma concolor coryi*), for instance, was genetically rescued by introducing individuals from a different subspecies [53]. Interestingly, the admixed Puma subspecies seemed to have genetic exchange via intermediate populations about 100 years beforehand [54] and its subspecific classification has been questioned by a genetic study [55]. Despite the existence of this and other promising examples of genetic rescue, admixing genetically different populations as a means of genetic rescue remains an underappreciated management tool [50].

## Supporting Information

S1 Table Information about the used microsatellite loci. (DOC)
