# Supplementary material for: Molecular Analyses Reveal Unexpected Genetic Structure in Iberian Ibex Populations
Source: PLoS One. 2017 Jan 30;12(1):e0170827. doi: 10.1371/journal.pone.0170827 (PMC5279733; doi:10.1371/journal.pone.0170827)
Supplement: S1 Table — (DOC) [file pone.0170827.s001.doc]

Table S1: Information about the used microsatellite loci

| FA | PCR | Ann. Temp. | locus | Chr[species] | Allele | GB acc.nr. | source |
| --- | --- | --- | --- | --- | --- | --- | --- |
| 1 | A | 57 | OarVH34 | 5 [*Capra hircus*] | 172, 174 | L12559 | [1] |
| 1 | A | 57 | BM4208 | 9 [*Bos taurus*] | 162, 164, 166, 168 | G18509 | [2] |
| 1 | A | 57 | MCM152 | 13 [*Bos taurus*] | 131, 133, 135, 137, 139 | L39825 | [3] |
| 1 | A | 57 | BM302 | 14 [*Bos taurus*] | 143, 145, 147, 149, 151 | G18774 | [2] |
| 1 | A | 57 | MAF209 | 17 [*Ovis aries*] | 104, 106 | M80358 | [4] |
| 2 | B | 59 | ILSTS30 | 2 [*Bos taurus*] | 165, 167, 169, 171, 173, 175 | L37212 | [5] |
| 2 | B | 59 | OarFCB20 | 2 [*Bos taurus*] | 191, 193, 195 | L20004 | [6] |
| 2 | B | 59 | SR-CRSP01 | NA [*Capra hircus*] | 123, 129, 133, 134, 139 | NA | [7] |
| 2 | B | 59 | BM1225 | 20 [*Bos taurus*] | 229, 239, 241, 243, 245, 247, 251, 259 | G18419 | [2] |
| 2 | B | 59 | IDVGA30 | 21 [*Bos taurus*] | 156, 158, 164, 176, 178 | X85049 | [8] |
| 2 | B | 59 | TGLA122 | 21 [*Bos taurus*] | 143, 145, 147, 149,151, 153 | NA | [9] |
| 2 | B | 59 | JMP29 | 24 [*Ovis aries*] | 122, 124, 128, 130 | U30893 | [10] |
| 2 | B | 59 | SR-CRSP24 | NA [*Capra hircus*] | 154, 156, 158, 160 | NA | [11] |
| 3 | C | 62 | ETH10 | 5 [*Capra hircus*] | 202, 208, 210, 212 | Z22739 | [12] |
| 3 | C | 62 | BM415 | 6 [*Bos taurus*] | 119, 121 | G18413 | [2] |
| 3 | C | 62 | OarFCB48 | 17 [*Capra hircus*] | 151 | M82875 | [6] |
| 3 | C | 62 | BM4505 | 26 [*Bos taurus*] | 269, 275, 277, 279, 287 | G18511 | [2] |
| 3 | C | 62 | MAF36 | 26 [*Bos taurus*] | 105, 109, 111, 113, 117, 119, 125, 127 | M80519 | [13] |
| 3 | C | 62 | OarKP6 | 3 [*Ovis aries*] | 180, 182, 184, 186 | AF223411 | [14] |
| 3 | C | 62 | SR-CRSP25 | NA [*Capra hircus*] | 100, 102, 106, 108, 110 | NA | [11] |
| 4 | E | 59 | ILSTS29 | 3 [*Capra hircus*] | 162, 170, 172, 174, 176, 178, 180, 184 | L37252 | [5] |
| 4 | E | 59 | CSSM47 | 8 [*Bos taurus*] | 138, 140, 142, 144, 148, 150 | U03821 | [15] |
| 4 | E | 59 | MILSTS076 | 9 [*Bos taurus*] | 118, 128, 130, 136, 138, 140 | 9982 | [5] |
| 4 | E | 59 | OARFCB193 | 19 [*Capra hircus*] | 121, 123, 125, 127, 131, 133 | L01533 | [16] |
| 5 | K | 55 | SR-CRSP07 | NA [*Capra hircus*] | 136, 138 | NA | [17] |
| 5 | K | 55 | TGLA10 | 8 [*Bos taurus*] | 177, 181, 183, 185 | NA | [18] |
| 5 | K | 55 | URB058 | 13 [*Bos taurus*] | 143, 159, 161, 163, 167 | U21788 | [19] |
| 5 | K | 55 | BM1258 | 23 [*Bos taurus*] | 190, 196, 198, 200, 202 | G18385 | [2] |
| 5 | K | 55 | BM1818 | 23 [*Bos taurus*] | 255, 257, 261 | G18391 | [2] |
| 5 | K | 55 | INRABERN175 | 25 [*Bos taurus*] | 138, 149, 151, 153, 155 | NA | [20] |

FA: fragment analysis run; PCR: multiplex reaction for PCR; Ann.temp: annealing temperature in PCR reaction; locus: name of microsatellite; Chr.[species]: location of the microsatellite for the given species; GB access.nr: Genbank accession number; reference: reference of the microsatellite

**Primer references**

1. Pierson CA, Hanrahan V, Ede AJ, Crawford AM. Ovine microsatellites at the OarVH34, OarVH41, OarVH58, OarVH61 and OarVH72 loci. Unpublished. 1993.

2. Bishop MD, Kappes SM, Keele JW, [Stone RT](https://www.ncbi.nlm.nih.gov/pubmed/?term=Stone RT%5BAuthor%5D&cauthor=true&cauthor_uid=7908653), [Sunden SL](https://www.ncbi.nlm.nih.gov/pubmed/?term=Sunden SL%5BAuthor%5D&cauthor=true&cauthor_uid=7908653), [Hawkins GA](https://www.ncbi.nlm.nih.gov/pubmed/?term=Hawkins GA%5BAuthor%5D&cauthor=true&cauthor_uid=7908653), et al. A Genetic-Linkage Map for Cattle. Genetics. 1994;136:619-39.

3. Davies KP, Maddox JF, Matthews P, Hulme DJ, Beh KJ. Ovine dinucleotide repeat polymorphism at the McM15, McM152, McM159, McM164 and McM210 loci. Animal Genetics. 1995;26:371.

4. Buchanan FC, Crawford AM. Ovine Dinucleotide Repeat Polymorphism At The Maf70 Locus. Animal Genetics. 1992;23:185.

5. Kemp SJ, Hishida O, Wambugu J, [Heyen DW](https://www.ncbi.nlm.nih.gov/pubmed/?term=Heyen DW%5BAuthor%5D&cauthor=true&cauthor_uid=8624035), [Beever JE](https://www.ncbi.nlm.nih.gov/pubmed/?term=Beever JE%5BAuthor%5D&cauthor=true&cauthor_uid=8624035), [Green CA](https://www.ncbi.nlm.nih.gov/pubmed/?term=Green CA%5BAuthor%5D&cauthor=true&cauthor_uid=8624035), et al. A Panel Of Polymorphic Bovine, Ovine And Caprine Microsatellite Markers. Animal Genetics. 26, 299-306.

6. Buchanan FC, Galloway SM, Crawford AM. Ovine Microsatellites At The Oarfcb5, Oarfcb19, Oarfcb20, Oarfcb48, Oarfcb129 And Oarfcb226 Loci. Animal Genetics. 1994;25:60.

7. Arevalo E, Holder DA, Derr JN, Bhebhe E, Linn RA, Ruvuna F, et al. Caprine Microsatellite Dinucleotide Repeat Polymorphisms At The Sr-Crsp-1, Sr-Crsp-2, Sr-Crsp-3, Sr-Crsp-4 And Sr-Crsp-5 Loci. Animal Genetics. 1994;25:202.

8. Mezzelani A, Zhang Y, Redaelli L, Castiglioni B, Leone P, Williams JL. et al. Chromosomal Localization And Molecular Characterization Of 53 Cosmid-Derived Bovine Microsatellites. Mammalian Genome. 1995;6:629-35.

9. Georges M, Massey JM. Polymorphic DNA markers in Bovidae, Patent WO 92/13102. Unpublished. 1992.

10. Penty JM, Lord EA, Montgomery GW. Characterisation and linkage mapping of ten sheep microsatellite markers derived from a sheep x hamster cell hybrid. Unpublished. 1995.

11. Yeh C, Kogi JK, Holder M, Guerra TM, Davis SK, Taylor JF. Caprine microsatellite dinucleotide repeat polymorphisms at the SR-CRSP 21, 22, 23, 24, 25, 26, and 27 loci. Animal Genetics. 1997;28:370-1.

12. Solinas TS, Fries R, Steffen P, Neibergs HL, Barendse W, Womack JE. et al. Physically mapped cosmid-derived microsatellite markers as anchor loci on bovine chromosomes. Mammalian Genome. 1993;720-7.

13. Swarbrick PA, Buchanan FC, Crawford AM. Ovine Dinucleotide Repeat Polymorphism At The Maf36 Locus. Animal Genetics*.* 1991;22:377-8.

14. Paterson KA, Crawford AM. Ovine microsatellite OarKP6 isolated from a BAC containing the ovine interferon gamma gene. Animal Genetics. 2000;31:343.

15. Moore SS, Byrne K, Berger KT, [Barendse W](https://www.ncbi.nlm.nih.gov/pubmed/?term=Barendse W%5BAuthor%5D&cauthor=true&cauthor_uid=8180478), [McCarthy F](https://www.ncbi.nlm.nih.gov/pubmed/?term=McCarthy F%5BAuthor%5D&cauthor=true&cauthor_uid=8180478), [Womack JE](https://www.ncbi.nlm.nih.gov/pubmed/?term=Womack JE%5BAuthor%5D&cauthor=true&cauthor_uid=8180478), et al. Characterization Of 65 Bovine Microsatellites. Mammalian Genome. 1994;5:84-90.

16. Buchanan FC, Crawford AM. Ovine microsatellites at the OarFCB11, OarFCB128, OarFCB193, OarFCB266 and OarFCB304 loci. Animal Genetics. 1993;24:145.

17. Bhebhe E, Kogi J, Holder DA, Arevalo E, Derr JN, Linn RA, et al. Caprine Microsatellite Dinucleotide Repeat Polymorphisms At The Sr-Crsp-6, Sr-Crsp-7, Sr-Crsp-8, Sr-Crsp-9 And Sr-Crsp-10 Loci. Animal Genetics. 1994;25:203.

18. Barendse W, Armitage SM, Kossarek LM, Shalom A, Kirkpatrick BW, Ryan AM, et al. A Genetic-Linkage Map Of The Bovine Genome. Nature Genetics. 1994;6:227-35.

19. Ma RZ, Russ I, Park C, Heyen DW, Beever JE, Green CA, et al. Isolation and characterization of 45 polymorphic microsatellites from the bovine genome. Animal Genetics. 1996;27:43-7.

20. Vaiman D, Schibler L, Bourgeois F*,* [Oustry A](https://www.ncbi.nlm.nih.gov/pubmed/?term=Oustry A%5BAuthor%5D&cauthor=true&cauthor_uid=8878693), [Amigues Y](https://www.ncbi.nlm.nih.gov/pubmed/?term=Amigues Y%5BAuthor%5D&cauthor=true&cauthor_uid=8878693), [Cribiu EP](https://www.ncbi.nlm.nih.gov/pubmed/?term=Cribiu EP%5BAuthor%5D&cauthor=true&cauthor_uid=8878693). et al. A genetic linkage map of the male goat genome. Genetics. 1996;144:279-305.
